# Dietary habits of patients with coronary artery disease in a tertiary-care hospital of Bangladesh: a case-controlled study

**DOI:** 10.1186/s41043-021-00226-1

**Published:** 2021-03-01

**Authors:** Taslima Khatun, Asirul Hoque, Kazi Selim Anwar, Manika Rani Sarker, Ferdous Ara, Dilara Maqbool

**Affiliations:** 1 Department of Public Health, College of Medicine, National Cheng Kung University (NCKU), Taiwan City, Taiwan; 2 Department of Community Nutrition, Faculty of Public Health, Bangladesh University of Health Sciences (BUHS), 125/1, Darus Salam, Mirpur, Dhaka, 1 Bangladesh; 3 Department of Community Nutrition, Faculty of Public Health, Bangladesh University of Health Sciences (BUHS), 125/1, Darus Salam, Mirpur, Dhaka 1, Bangladesh; 4 Infectious Diseases Department, International University of Health & Welfare (IUHW), Narita, Japan; 5 Department of Community Nutrition, Faculty of Public Health, Bangladesh University of Health Sciences (BUHS), 125/1, Darus Salam, Mirpur, Dhaka 1, Bangladesh; 6 Department of Food and Nutrition, AkijCollege of Home Economics, Road 9/A (New), House118, Dhanmondi, Dhaka, 1209 Bangladesh; 7 Nutrition Officer, LabAid Cardiac Hospital, Dhaka, 1205 Bangladesh

**Keywords:** Coronary arterial diseases, Dietary intake, CVD-risk factors, Bangladesh

## Abstract

**Background and objectives:**

Globally, coronary artery disease (CAD) remains one of the leading causes of death, both in developed and less economically developed countries (LEDC) including Bangladesh. Diet plays a key role in the pathogenesis processes of atherosclerosis and coronary artery disease (CAD). The purpose of this study was to assess the dietary habit of heart disease cases that had CAD against matched controls.

**Methodology:**

Complying Helsinki ethical norms, with written consent, this case-control study was performed among 210 subjects: 105 CAD-hospitalized patients (selected from Lab Aid Cardiac and Specialized Hospitals) and 105 healthy subjects from local urban communities having their body mass index (BMI: ranging between ≥18.5 and 27 socio-demographic status, detailed-dietary patterns and blood pressure levels were recorded, anthropometric indices measured, and serum biochemistry (complete lipid profile) tested/analyzed for both the cases and controls. All visually re-checked data were analyzed using appropriate statistical tools (*t* test/conditional-logistic regressions) on SPS/Windows V.21.0.

**Result:**

Almost half (45%) CAD patients had hypertriglyceridemia and higher levels of low-density lipoprotein, significantly higher BMA (*p*=0.001), waist circumference, and waist to hip ratio in male patients (*p*=0.005 and *p*=0.020, respectively) than their peer controls. Serum lipid profiles, sugar concentrations, and blood pressure levels of CAD patients revealed higher levels than clinically defined cut-off values as established risk factors for CAD. Odds ratios (CI 95%) as risk factors for consuming junk food {OR=5.49 (2.25–13.38)}, chicken {OR=4.54 (1.89–10.9) was the most, followed by beef {OR=2.68 (1.19–4.98)}, eggs {OR=2.38 (1.14–10.92)}, fish {OR=2.81 (1.31–6.04)}, and vegetables {0R=.968 (0.510–1.839)}. However, fat-free milk, ghee/butter oil, curd/yogurt, and fruits had lower ORs revealing no or less risks for CAD.

**Conclusion:**

Food habits of CAD patients (with higher BMI level and biochemical indicators of the blood) statistically revealed that consuming junk food, meat, and eggs being riskier, fruits, fat-free milk, yogurt, and vegetable remains have protective effects on CAD.

## Background

Coronary artery disease (CAD) is the leading cause of death, globally. It is one of the most common chronic illnesses in the developing world [1, 2]. According to the World Health Organization, the number of deaths due to ischemic heart disease (IHD) in 2001 was 24, 84, 000 in developing countries compared to 3, 512, 000 in the Less Economically Developed Country (LEDC). The focus for dietary recommendations in Bangladesh are as follows: a balanced diet, eat a variety of foods from each of the food groups, and improve dietary diversity and quality as well as wise food selection in planning meals [3].

The historical advice to reduce the consumption of saturated fat was challenged by recent studies, including meta-analyses of prospective studies [ 4, 5], which led the UK Scientific Advisory Committee on Nutrition (SACN) [ 6] and the WHO [7] to conclude, based on evidence from both randomized, controlled trials, and prospective cohort studies, that the saturated fat intake is not associated with cardiovascular disease-related mortality. Although most information on the nutritional risk factors CVD is derived (not clear) from the western reports, the prevalence of CVD is rapidly evolving towards epidemic proportions in the LEDCs with rapid changes in people’s lifestyle and food consumption behavior over the past few years [8, 9]^.^However, a study in the USA did not find a significant association between dietary intake of eggs and all-cause and heart disease mortality among the US adults, whereas the total dietary cholesterol intake at high levels seemed to be associated with the higher risk of all-cause mortality [10].

Based on the aforementioned facts and data, one can logically postulate that the absolute risk assessment provides a more accurate estimate of overall, individualized CAD risk as well. There is no sufficient number of published reports on the relationship between the food intake patterns and the risk of developing CAD in Bangladesh; we thus undertook a study to assess the dietary habit of the people with heart diseases (CAD) against their age, sex, and socioeconomic matched controls.

## Methodology

### Enrollment of patients

This case-control study was conducted at the LabAid Cardiac Hospital and LabAid Specialized Hospital in Dhaka. Of the 210 subjects, 105 randomly selected CAD patients (53 males and 52 female) were studied against 105 ages (40–75 years) and sex-matched healthy controls (54 male and 51females) that had a BMI ranging from ≥18.5 to 27.5. The subjects were given a prescheduled time for data collection. The cases were randomly selected from the OPD of the LabAid Cardiac Hospital and their medical record, identified as a CAD patient. On the other hand, control patients were the matched healthy subjects from the same hospital who visited it for a routine check-up; they were identified as non-CAD patients.

### Data collection

With prior written consents of the subjects who agreed to take part in the study after explaining them the nature and purpose of the study, they were, however, clearly briefed the potential risks of all the procedures used in the study. Both the CAD patients and the healthy controls were interviewed using a pre-tested semi-structured questionnaire, and then, the I.V. blood samples were collected. Data and blood samples of the healthy controls were collected while they were at the residence. However, data and blood samples of the cases (CAD patients) were collected from the IPD of the two LabAID and Cardiac Hospitals. The non-response rate among the cases was 10% (11/105), and it was ~5% (5/105) among the control group.

### Anthropometric measurements

Body weights were measured in light clothing, without shoes to the nearest 0.1 kg using a digital balance. Heights were measured to the nearest 0.1 cm using the SECA balance with height attachment. Measurement of the waist circumference as a surrogate for visceral adipose tissue was performed in the horizontal plane above the iliac crest [9]. BMI cut-offs for Asians and Indians proposed by the International Obesity Task Force (IOTF-2000) were used for identifying over weight and obesity which were; <18.49 kg/m^2^ for underweight, 18.5 to 22.9 kg/m^2^ for normal weight, 23 to 27.49 kg/m^2^ for overweight, and >27.50 kg/m^2^ which indicates obese [11].

### Assessment of food habits

Nutritional evaluation was accomplished using a customized Harvard food-frequency questionnaire that includes 57 food items mainly consumed by individuals daily and weekly over the past 7 days [12]. The frequency of consumption was semi-quantified in terms of the number of each food items consumed in a day or week [13].

### Clinical assessment of CAD patients

Blood pressure of each patient was measured in the right arm (average of 3 measurements in the seated position while resting and not tired, over whelmed, or anxious). A blood pressure greater than or equal to140/90 mmHg or on prescribed antihypertensive medication(s) was classified as hypertensive [14].

Interviews were conducted during the first 72 h of hospitalization for the case group. The past medical histories of individuals and their reports assisted us in characterizing them as having hypertension, hypercholesterolemia, or diabetes.

### Laboratory tests/methods (in short) and essential calculations

Serum glucose (FBS) was measured using glucose–oxidase method, and the fasting serum lipid profile (cholesterol, triglyceride, and HDL) was determined by enzymatic colorimetric method. Serum LDL was calculated using the formula of Friede wald [15]. Hypercholesterolemia and hypertriglyceridemia were defined as serum total cholesterol (TC) and triglycerides (TG) levels of >200 and 150 mg/dL, respectively, or if hypo-lipidemic treatment was administered. Diabetics were those with fasting blood glucose equal to or greater than 126 mg/dL for two times or those who were under diabetic diet or medications.

### Statistical analysis

All the collected and visually and logically rechecked data were analyzed using the SPSS software (Windows.V.21.5) using appropriate statistical lines. Estimations on the effect of each food item were performed using odds ratio (OR) at 95% confidence intervals (95% CI) employing binary logistic regression analysis, adjusting for age, BMI, smoking, history of hypercholesterolemia, hypertension, or diabetes. *t* test also done to compare means among two groups. All the *p* values were taken from two-sided tests at a significant level of 5%.

## Results

The mean age of the patients was 51.5±9.0 and of the controls was 50.8±9.2 years, revealing no significant difference (*p*>0.43), a like sex (*p* value for the male patients was >0.49 and the that of females was >0.47). Similarly, no differences were found in their basic nutritional characteristics between the cases and the controls: in terms of BMI (*p*<0.001), the WC for males was (*p*=0.005) and females (*p*=0.558) while the WHR was (*p*=0.020 and 0.125 for males and females, respectively) (Table 1). Contrarily, all of the comparative values in biochemical parameters of the cases differed significantly compared to those of the matched controls, except FBS (6.72±2.39 vs. 6.56±2.80, *p*>0.67) and diastolic-BP (74.51±12.6 vs. 77.0±9.4, *p*>0.12). Thus, the values of the total S. cholesterol among the CAD patients (203.5±58.2) and those of the control patients (155.93±53.88), differed significantly (*p*≤0.001), alike LDL (132.15±49.72 vs. 95.25±32.44 (*p*≤0.001), triglyceride {128.50 (54–780) vs. 120.28±58.18} (*p*=0.001), HDL (38.23±7.46 and 44.7±8.41) (*p*≤0.001), and systolic blood pressure (118.55±17.64 and 127.71±11.96) (*p*≤0.001) (Table 2). Similarly, while assessing the important pre-fixed risk factors, hypercholesterolaemia and hypertriglyceridaemia were higher in the CAD patients than those in the controls with *p*≤0.001 and p=0.001, respectively. The total cholesterol (TC) and HDL were strongly significant (*p*≤0.001) including LDL (*p*<0.028) in the CAD patients than in the controls. The lipid profiles among both the case sand the controls had higher than its cut-off values for risk factors. Systolic blood pressure (0.022) was also significantly higher among the CAD patients (Table 3).

**Table 1:** Basic characteristics and anthropometric parameters of CAD patients and Controls (*n*=210)

Variables	CAD patients	Controls	***p*** value
**Age (year) M±SD**	55.21 ±.8.94	56.20±8.88	
**Male (%)**	53	54	0.487
**Female (%)**	52	51	0.468
**BMI (kg/m**^**2**^**)**	25.12±2.18	24.22±2.19	0.004
**WC (cm, male)**	92.06±7.4	88.10±6.3	0.005
**WC (cm, female)**	86.86±8.32	85.79±9.3	0.558
**WHR (male)**	0.96±0.053	0.94±0.035	0.020
**WHR (female)**	0.96±0.082	0.94±0.059	0.125

Results were expressed as number and mean ±SD with *t* test performed for the analysis; *p*<0.05 is considered; BMI body mass index, *WHR* waist hip ratio, *WC* waist circumference

**Table 2:** Biochemical parameter of the study respondents (*n*=210)

Biochemical parameters	CAD patients (M±SD)	Controls (M±SD)	***p*** value
**Fasting blood sugar (**mmol/l**)**	6.72±2.39	6.56±2.80	0.658
**Total blood cholesterol (**mg/dl**)**	203.45±58.15	155.93±53.88	<0.001
**LDL (**mg/dl**)**	132.15±49.72	95.25±32.44	<0.001
**Triglyceride (**mg/dl**)**	128.50 (54-780)	100.00 (50-350)	*0.001
**HDL (**mg/dl**)**	38.23±7.46	44.7±8.41	<0.001
**Systolic blood pressure (**mm hg)	118.55±17.64	127.71±11.96	<0.001
**Diastolic blood pressure (**mm hg)	74.51±12.56	77.00±9.43	0.115

Results were expressed as mean ± SD and median (range). Independent samples *t* test and *Mann-Whitney test were performed as test of significance (*p*<0.05); *LDL* low-density lipoprotein, *HDL* high-density lipoprotein

**Table 3:** Comparison of selected cardiovascular disease risk factors between CAD patients and controls

Variable	CAD patients (***n***=105)	Controls (***n***=105)	***p*** value
TG > 150 mg/dl	45 (43)	23 (22)	0.001
TC >200 mg/dl	59 (59)	31 (30)	<0.001
LDL-C >100 mg/dl	71(68)	40 (38)	0.028
HDL-C < 35 mg/dl	68 (65)	28 (27)	<0.001
FBS > 126 mg/dl	44 (42)	39 (37)	0.283
BP >140/90 mm hg	52 (50)	35(33)	0.022
BMI>27.5 kg/m^2^	63 (60)	33(31)	<0.001
Cigarette smoking habit	26 (25)	10 (10)	<0.001

*Abbreviations: TG* triglyceride, *TC* total cholesterol, *FBS* fasting blood sugar, *BP* blood pressure, *LDL* low-density lipoprotein, *HDL* high-density lipoprotein

Binary logistic regression analysis was performed against consumption of different food items beef (>2 times/week), chicken (> 2 times/week), egg (> 2 times/week), fish (< 2 times/week), junk foods (burger, pizza, pastry, etc.) (> 2 times/week), fruits and vegetable (< 2 times/day) by adjusting for age, BMI, smoking, history of hypercholesterolemia, hypertension, and diabetes. Significant association was found for beef, chicken, egg, fish, junk foods, fruits, and vegetables [OR (95%CI){2.68(1.19–4.98), *p*=0.017},{4.54 (1.89–10.94), *p*=0.001},{2.38 (1.14–10.92) *p*=0.021},{2.81 (1.31–6.04), *p*=0.008}, {5.49 (2.25–13.38), *p*≤0.001}, {0.580 (0.311–1.083), *p*=0.087}, and {0.968 (0.510–1.839), *p*=0.921}], respectively, with CAD patients and controls. No association was found between daily consumption of fat-free milk and weekly consumption of ghee/butter-oil and curd/yogurt with risk of CAD. In the unadjusted model, beef, chicken, egg, fish, junk foods, fruits, and vegetables were found significantly associated with the risk of CAD; [OR (95%CI) {0.386 (0.171–0.872), *p*=0.022}, {0.269 (0.110–0.659), *p*=0.004}, {0.476 (0.225–1.004), *p*=0.051}, {0.312 (0.140–0.692), *p*=0.004}, {0.189 (0.077–0.464), *p*≤0.001}, {0.428 (0.198–0.925), *p*=0.031}, and{0.372 (0.174–0.795), *p*= 0.011}] (Table 4).

**Table 4:** Binary logistic regression analysis of dietary intake pattern with CAD patients and controls

Food groups	Consumption frequency	CAD patients ***n***=105	Controls ***n***=105	Adjusted values				Unadjusted values			
				Odds ratio	95% C.I.		***p*** value	Odds ratio	95% C.I.		p value
					Lower	Upper			Lower	Upper	
Beef	> 2 times/week	35	15	2.681	1.192	4.980	0.017	0.386	0.171	0.872	0.022
Chicken	> 2 times/week	37	10	4.545	1.891	10.94	0.001	0.269	0.110	0.659	0.004
Egg	> 2 times/week	63	53	2.381	1.141	10.92	0.021	0.476	0.225	1.004	0.051
Fish	< 2 times/week	52	82	2.816	1.311	6.047	0.008	0.312	0.140	0.692	0.004
milk	< 2 times/week	65	59	1.632	0.612	3.457	0.224	1.370	0.657	2.856	0.401
Ghee	> 2 times/week	57	41	1.625	0.799	3.304	0.180	0.628	0.305	1.297	0.209
Curd	> 2 times/week	67	85	1.682	0.711	3.977	0.237	0.496	0.204	1.209	0.123
Junk food	> 2 times/week	37	10	5.496	2.257	13.381	<0.001	0.189	0.077	0.464	<0.001
Fruits	< 2 time/day	52	71	0.580	0.311	1.083	0.087	0.428	0.198	0.925	0.031
Vegetables	< 2 times/day	54	74	0.968	0.510	1.839	0.921	0.372	0.174	0.795	0.011

Results were expressed as number with odds ratio performed for the analysis; *p*<0.05 is considered as statistically significant and 95% confidence interval

## Discussion

Most cardiovascular disease (CVD) occurs in the presence of traditional risk factors, including hypertension and dyslipidemia, and these, in turn, are influenced by behavioral factors, such as dietary habit and lifestyle. According to the BMI levels as suggested by the WHO [16], we observed that more CAD patients belonged to the high-risk groups (BMI>27.5 kg/m^2^) compared to their controls. Waist circumferences were also higher among the CAD patients than among their counterparts. It has been observed that BMI and WHR were significantly lower among the people with healthy food habit which might support the dietary recommendations for a healthy weight [16]. The dietary factors are, therefore, vital for the development of obesity and cardiovascular disease (CVD) [17]. In our study, we found that cardiovascular risk factors, such as total cholesterol, triglyceride, LDL, HDL, and high blood pressure were strongly related to CAD. Hypertriglyceridemia and high levels of LDL-C were prevalent most CHD patients (99%) while half of the control patients showed hypertriglyceridemia and one-fifth had high concentrations of LDL-C [18]. However, many issues remain unsettled, and we need additional knowledge on the optimal level of intake of nutrients and foods and how the intake varies between individuals. The intake of energy and a few nutrients at intervals has demonstrated a higher risk for developing angina pectoris, myocardial infarction [19], and CVD-related mortality [20]. Two prospective studies in (Framingham, Massachusetts and Baltimore, Maryland) found that the healthy food pattern is protective against overweight [21]and prevents the increase in both BMI and waist circumference [22]as a potential risk factor for developing CAD. A significant association was found in the weekly and some daily consumption of foods with a risk of CAD.

Logistic regression analysis was performed against the confounding independent variables. On the other hand, no association was found between the weekly consumption of ghee and curd and the daily consumption of milk with the risk of CAD. Some studies reported that a healthier eating pattern is associated with consuming medically prescribed diet [19, 23], reporting changed food habits [24, 25]. According to a review by Giugliano et al. [25], many diets high in refined starch, sugar, and saturated and trans fat and low inn–3 fatty acids and natural antioxidants and fiber from fruit, vegetables, and whole grains cause the inflammation associated with the metabolic syndrome by affecting the immune system. It has been reported that the consumption of fruits and vegetables three or more servings a day versus less than once a day is associated with a 27%t reduction in cardiovascular disease risk [26]. Despite achieving a reduction in the total fat intake between the intervention and the control groups (28.8% vs. 37%t of calories (*p*<0.001), respectively), no significant effects of the intervention on CHD, stroke, or CVD were observed during the 8-year follow-up [27]. Although fish has a number of important nutritive qualities, their major cardiovascular benefit likely derives from their content of the omega-3 fatty acids, eicosapentaenoic acid (EPA), and docosahexanoic acid (DHA) [28] This effect appears to be related to enrichment of membrane phospholipids with omega-3 fatty acids and a resulting reduction in risk for abnormal cardiac electrical conductivity [29]. Other properties of these fatty acids that may benefit from the risk for CAD include anti-platelet aggregation and anti-inflammatory effects and also reduction in plasma triglycerides at higher doses [30].

The prospective cohorts study found that there was associations between macronutrient intakes and health (mortality and CVD risk) are non-linear [31]. One of the largest studies was conducted among 177,000 people in 50 countries, and they did not found any significant association between egg intake and blood lipids, mortality, or major CVD events [32].

To summarize, our findings suggest that the consumption of healthy food develop better anthropometric feature, keeping blood pressure and maintaining blood lipid values within the normal limit compared to other unhealthy dietary patterns. The group of people with this healthy dietary pattern may be distinguished from the population in general by more frequent consumption of high-fiber cereals, low-fat milk products, fruits, vegetables, fish, and less frequent consumption of products rich in fat and sugar.

A probable limitation of this study is the lack of data on portion sizes in the FFQ. The participants’ dietary intake was assessed using a questionnaire which did not take into account the intake grams of foods; resulting lack of adjustment for the confounding effect of *r* potential nutrients intake in association analyses between the dietary patterns and the dependent variables in our study

## Conclusions

Anthropometric parameters, such as BMI, WC, and WHR, are significantly associated with the CAD risk. Consumption of junk foods is associated with a significantly higher risk of coronary artery diseases. On the contrary, consumption of fish, fruits, fresh vegetables, and fat-free yogurt has the protective effects on CAD while beef and egg have a role to increase the risk of CAD. There is a great need to conduct more study is on dietary risk factors to explore the risk of cardiac diseases with consumption of different compositions of food and portion size. It is clear that nutrition-related cardiac risk factor should be emphasized for the prevention of coronary artery disease in our country.

## Data Availability

Please contact author for data requests.
