# Correction to: Dietary habits of patients with coronary artery disease in a tertiary-care hospital of Bangladesh: a case-controlled study

**DOI:** 10.1186/s41043-021-00243-0

**Published:** 2021-04-13

**Authors:** Taslima Khatun, Dilara Maqbool, Ferdous Ara, Manika Rani Sarker, Kazi Selim Anwar, Asirul Hoque

**Affiliations:** 1 Department of Public Health, College of Medicine, National Cheng Kung University (NCKU), Taiwan City, Taiwan; 2 Department of Community Nutrition, Faculty of Public Health, Bangladesh University of Health Sciences (BUHS), 125/1, Darus Salam, Mirpur, Dhaka, 1 Bangladesh; 3 Department of Community Nutrition, Faculty of Public Health, Bangladesh University of Health Sciences (BUHS), 125/1, Darus Salam, Mirpur, Dhaka, 1 Bangladesh; 4 Infectious Diseases Department, International University of Health & Welfare (IUHW), Narita, Japan; 5 Department of Community Nutrition, Faculty of Public Health, Bangladesh University of Health Sciences (BUHS), 125/1, Darus Salam, Mirpur, Dhaka, 1 Bangladesh; 6 Department of Food and Nutrition, AkijCollege of Home Economics, Road 9/A (New), House118, Dhanmondi, Dhaka, 1209 Bangladesh; 7 Nutrition Officer, LabAid Cardiac Hospital, Dhaka, 1205 Bangladesh

**Correction to: J Health Popul Nutr 40, 3 (2021)**

**https://doi.org/10.1186/s41043-021-00226-1**

Following publication of the original article [1], the authors reported an error in the order of the authors was not the order as it was originally accepted in. The correct order of the authors is indicated hereafter and the changes have been highlighted in **bold typeface**.

The incorrect order of the author reads:

Taslima Khatun, Asirul Hoque, Kazi Selim Anwar, Manika Rani Sarker, Ferdous Ara and Dilara Maqbool

The correct order of the authors should read:

**Taslima Khatun, Dilara Maqbool, Ferdous Ara, Manika Rani Sarker, Kazi Selim Anwar, Asirul Hoque**

The changes requested are implemented in this correction and the original article [1] has been corrected.
